# Supplementary material for: Effects of Nutritional Restriction during Laying Period of Fat and Lean Line Broiler Breeder Hens on Meat Quality Traits of Offspring
Source: Animals (Basel). 2021 Aug 18;11(8):2434. doi: 10.3390/ani11082434 (PMC8388661; doi:10.3390/ani11082434)
Supplement: Supplementary file 1 [file animals-11-02434-s001.zip › animals-1347154-supplementary.pdf]

Table S1. Effect of feed intake on sarcomere length and myofibril diameter of 56-day-old offspring in broiler hens during laying period.

Item	Line		Intake		Line × Intake				Pooled	*p*-value		
	L	F	NI	RI	LN	LR	FN	FR	SEM	Line	Intake	Line × Intake
**Sarcomere Length [1]**												
Chest Muscle	2.15	2.18	2.16	2.17	2.14	2.16	2.18	2.18	0.038	0.293	0.753	0.576
Thigh Muscle	2.20	2.20	2.19	2.22	2.16	2.25	2.23	2.18	0.085	0.986	0.690	0.229
**Myofibril Diameter [1]**												
Chest Muscle	1.11	1.12	1.08	1.15	1.09	1.14	1.08	1.17	0.060	0.805	0.103	0.671
Thigh Muscle	1.01	1.07	1.05	1.03	1.01	1.01	1.09	1.04	0.061	0.173	0.535	0.637

Abbreviations: L, lean line; F, fat lean; NI, normal daily feed intake; RI, restricted daily feed intake; LN, lean line with normal daily feed intake; LR, lean line with restricted daily feed intake; FN, fat line with normal daily feed intake; FR, fat line with restricted daily feed intake; SEM, standard error of the mean. [1] the unit for sarcomere length and myofibril diameter was μm.
